# Effect of Belimumab on the Cutaneous Manifestations of Patients With Lupus Erythematosus

**DOI:** 10.1111/ijd.70251

**Published:** 2026-01-22

**Authors:** Diana Schellin, Sarah Rösing, Nick Zimmermann, Nicolai Leuchten, Martin Aringer, Claudia Günther

**Affiliations:** ^1^ Department of Dermatology University Hospital Carl Gustav Carus Dresden, Technische Universität Dresden Dresden Germany; ^2^ Department of Dermatology and Venereology Helios Hospital Erfurt Erfurt Germany; ^3^ Division of Rheumatology, Department of Medicine III University Hospital Carl Gustav Carus, Technische Universität Dresden Dresden Germany; ^4^ Department of Dermatology University Hospital, Eberhard Karls University Tübingen Tübingen Germany

**Keywords:** belimumab, CLASI, cutaneous, long‐term, lupus erythematosus, RCLASI, skin

## Abstract

**Background:**

The aim was to assess the long‐term effect of belimumab on the cutaneous manifestations of patients with systemic lupus erythematosus (SLE).

**Methods:**

The retrospective analysis included 29 patients with SLE. Cutaneous disease activity was assessed using the Cutaneous Lupus Erythematosus Disease Area and Severity Index (CLASI) and the Revised CLASI (RCLASI) activity scores before treatment with belimumab and at 3, 6, 12, and up to 60 months of treatment. Eleven patients were treated for the whole observation period; however, only 8 patients were evaluable at 48 months, and only 6 at 60 months due to loss of follow‐up.

**Results:**

Compared to the baseline score, a significant reduction of the CLASI and RCLASI activity score was seen during the treatment (mean CLASI baseline 8.76 ± 5.12, after 3 months 5.59 ± 3.87, *p* < 0.001, after 60 months 4.67 ± 3.86, *p* = 0.017; mean RCLASI baseline 10.93 ± 6.57, after 3 months 7.62 ± 6.03, after 60 months 5.33 ± 4.31, *p* = 0.012). The percentage of patients achieving a CLASI 100 response increased continuously over time, reaching a maximum of 30% after 60 months. Response was most remarkable among patients with acute or discoid cutaneous lupus. The treatment was well tolerated by the patients, with the most common adverse events being respiratory infections, headaches, and arthralgia, among others.

**Conclusion:**

This retrospective analysis demonstrated that belimumab has long‐term benefits for cutaneous manifestations of patients with lupus erythematosus. However, prospective studies in larger patient populations are needed to confirm the findings.

## Key Points

- Belimumab has been shown to provide long‐term benefits for cutaneous manifestations in patients with lupus erythematosus.
- Response was most remarkable among patients with acute or discoid cutaneous lupus.
- The patients tolerated both short‐term and long‐term treatments with belimumab well.

## 1 Introduction

Systemic lupus erythematosus (SLE) is a chronic inflammatory autoimmune disease. The skin of patients with SLE is very frequently affected. These cutaneous manifestations have a broad spectrum, ranging from acute to subacute to chronic, and include non‐specific lupus lesions, such as vasculitis [1].

Cutaneous lupus manifestations can be very painful, mutilating, and stigmatizing, often representing a great psychological burden for the patients [2]. Even maximal standard therapy can sometimes not adequately control the disease [3]. Biologics such as belimumab can reduce cutaneous lesions but are currently only approved for antibody‐positive SLE [4].

Belimumab is a human IgG1 antibody that blocks the soluble B‐lymphocyte stimulator protein (BLyS), also known as BAFF, thereby preventing BLyS from binding to its receptor on B cells [5]. In the pivotal studies of belimumab (BLISS‐52, BLISS‐76), positive effects on the skin were observed, but no validated activity scores for skin lesions were used [6]. There are still very few retrospective studies on the effects of belimumab explicitly on the skin [7, 8]. In particular, long‐term results in a real‐world setting are rare. Therefore, this analysis aimed to examine the effect of belimumab on cutaneous manifestations in SLE patients.

## 2 Methods

### 2.1 Analysis and Design

A total of 29 patients with SLE were included in the retrospective analysis. All patients were recruited from the Department of Dermatology and the Division of Rheumatology, Department of Medicine III, at the Center for Autoimmune and Rheumatic Diseases, University Medical Centre Dresden, Germany. Patients gave their informed consent, and ethical approval was obtained. The patients were either given 200 mg belimumab subcutanously per week or intravenously in a dosage of 10 mg/kg body weight on Days 0, 14, 28, and every 4 weeks after that. All of the 29 patients were treated with belimumab for at least 6 months. The patients' skin was assessed using the Cutaneous Lupus Erythematosus Disease Area and Severity Index (CLASI) and the Revised CLASI (RCLASI) scores before treatment with belimumab and at 3, 6, 12, 24, 36, 48, and 60 months after treatment. Eleven patients were treated for 60 months, but only 8 patients were evaluable at 48 months and only 6 at 60 months due to loss of follow‐up.

### 2.2 Patient Population

The inclusion criteria were clinically and/or histologically confirmed lupus erythematosus (EULAR/ACR criteria), age over 18 years, and active cutaneous manifestations of lupus erythematosus (CLASI ≥ 2). Patients were excluded if the following criteria were met: treatment with belimumab for less than 3 months, no consistent description of skin activity in the patient file, non‐compliant patients, or patients with lupus erythematosus but without skin involvement. A patient flowchart is shown in Figure S1. The key clinical and demographic characteristics of the patients are presented in Table 1.

**TABLE 1:** Key clinical and demographic characteristics of the patients before treatment with belimumab.

Total	29 (100)
Gender, *n* (%)	
Female	28 (97)
Male	1 (3)
Age, years	
Mean (SD)	41.3 (12.2)
Median (range)	39.0 (25.0–74.0)
Duration of disease	
Mean (SD), years	9.9 (10.7)
Median (range), years	5.0 (1.0–47.0)
≤ 5 years, *n* (%)	15 (51.7)
> 5 years, *n* (%)	14 (48.3)
LE subtype, *n* (%)	
Acute cutaneous LE Subacute cutaneous LE Chronic discoid LE Non‐specific skin lesions in LE	3 (10.3) 6 (20.7) 11 (37.9) 9 (31.0)
CLASI activity	
Mean (SD)	8.76 (5.12)
Median (range)	8 (2–28)
RCLASI activity	
Mean (SD)	10.93 (6.57)
Median (range)	10 (1–28)
Severity*, *n* (%)	
Mild	20 (68.97)
Moderate to severe	9 (31.03)

Abbreviations: CLASI, Cutaneous Lupus Erythematosus Disease Area and Severity Index; LE, lupus erythematosus; RCLASI, Revised Cutaneous Lupus Erythematosus Disease Area and Severity Index; SD, standard deviation.

* mild = CLASI activity 0–9, moderate = CLASI activity 10–20, severe = CLASI activity ≥ 21.

### 2.3 Measurement Instruments

In the present analysis, the CLASI activity score [9, 10], was used as a measurement tool for assessing skin lesions and was measured prior to treatment with belimumab.

The Revised Cutaneous Lupus Erythematosus Disease Area and Severity Index (RCLASI) is a revised score of the CLASI. It enables a more detailed assessment of rare CLE subtypes [11]. Both the CLASI and the RCLASI activity scores were used in this analysis as measurement instruments for the therapeutic response.

### 2.4 Immunohistochemistry

Skin tissue samples were deparaffinized and incubated with the rat anti‐human BAFF antibody (Novus Biologicals, NBP1‐97622) for 60 min at room temperature. As a secondary antibody, goat anti‐rat IgM HRP (ThermoFisher, 31,476) was used for 20 min at room temperature. Thereafter, the slides were washed with PBS and stained with ImmPACT DAB Peroxidase Substrate (VectorLabs). The slides were then incubated in Mayer's hemalum solution (Engelbrecht Medizin‐und Labortechnik GmbH) for 50 s. After dehydration, the slides were mounted in CellTexx mounting medium (Engelbrecht Medizin‐und Labortechnik GmbH) and analyzed with a microscope. Scoring was defined as 3 for intense staining, 2 for medium staining, and 1 for low staining intensity. The findings were validated by two independent and blinded investigators through the analysis of staining of different CLE lesions before and after therapy.

### 2.5 Statistical Analysis

Data were tested for normal distribution by the Shapiro–Wilk test. Means and standard deviations of the CLASI and RCLASI activity scores were calculated for the time points *t* = 0, 3, 6, 12, 24, 36, 48, and 60 months, and then compared to the respective mean value at *t* = 0. Afterward, the ANOVA was used due to multiple measurement time points. In all performed tests, values of *p* ≤ 0.05 were considered statistically significant. As a post hoc test, Bonferroni's least significant difference was used.

## 3 Results

### 3.1 Cutaneous Lesions Improve During Treatment With Belimumab

**FIGURE 1:**
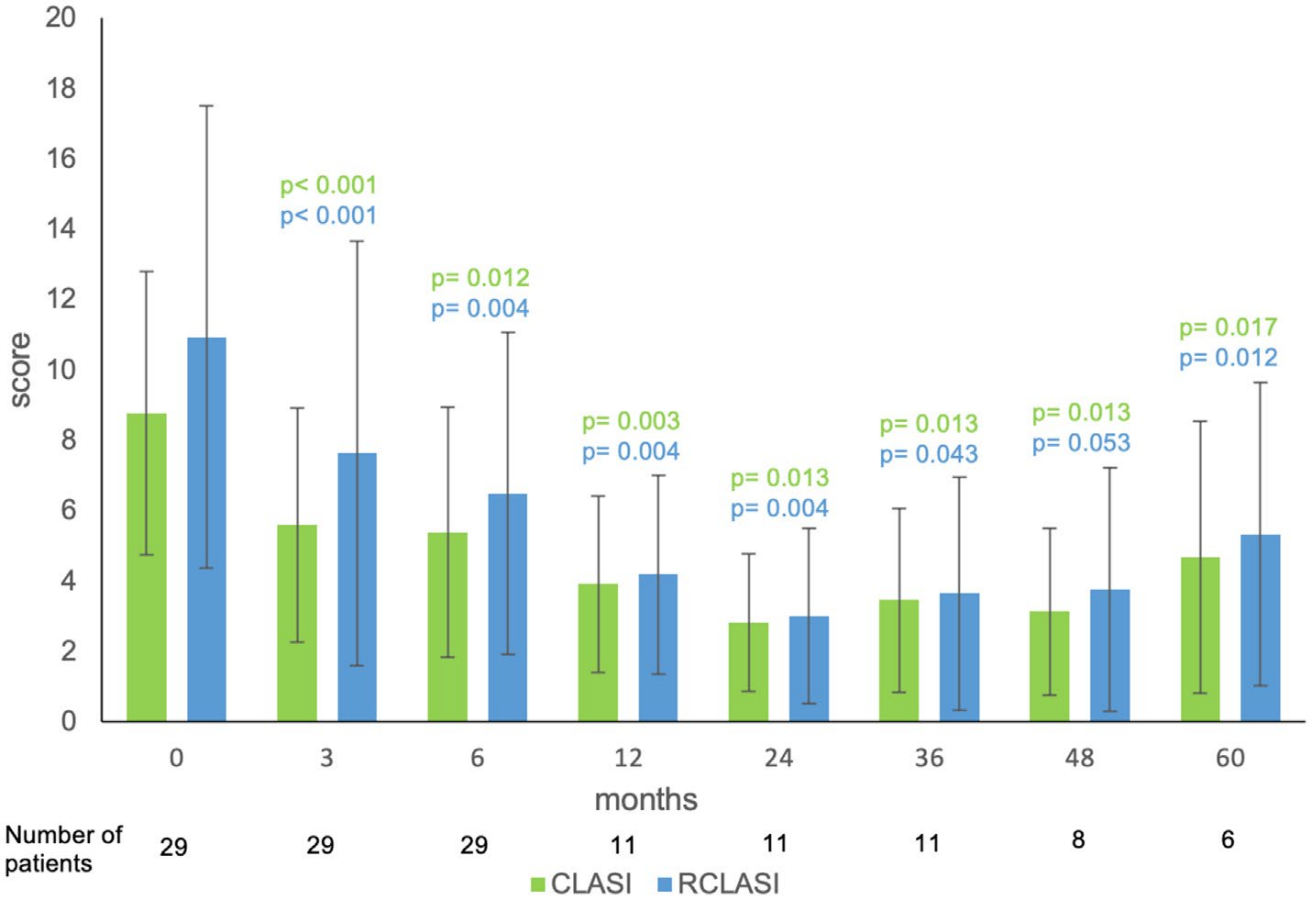
CLASI and RCLASI activity scores before and during treatment with belimumab. The means and SD are demonstrated. The number of patients is indicated. Differences were tested against time point 0 before treatment and assessed by ANOVA.

Patients with uncontrolled SLE and cutaneous involvement were treated with belimumab and had their CLASI scores routinely assessed every 3 months for 1 year and annually thereafter. During the treatment, the CLASI activity score decreased significantly at all measured time points. Mean CLASI before treatment was 8.76, and RCLASI was 10.93. By 3 months, CLASI and RCLASI declined to 5.59 (*p* < 0.001) and 7.62 (*p* < 0.001), respectively, after 6 months to 5.38 (*p* = 0.012) and 6.48 (*p* = 0.004), respectively, after 12 months to 3.91 (*p* = 0.003) and 4.18 (*p* = 0.004), respectively, after 24 months to 2.82 (*p* = 0.013) and 3.00 (*p* = 0.004), respectively, after 36 months to 3.45 (*p* = 0.013) and 3.64 (*p* = 0.043), respectively, after 48 months to 3.13 (*p* = 0.013) and 3.75 (*p* = 0.053), respectively, and after 60 months to 4.67 (*p* = 0.017) and 5.33 (*p* = 0.012), respectively. The time course of the CLASI and RCLASI activity score during the treatment is shown in Figure 1.

**FIGURE 2:**
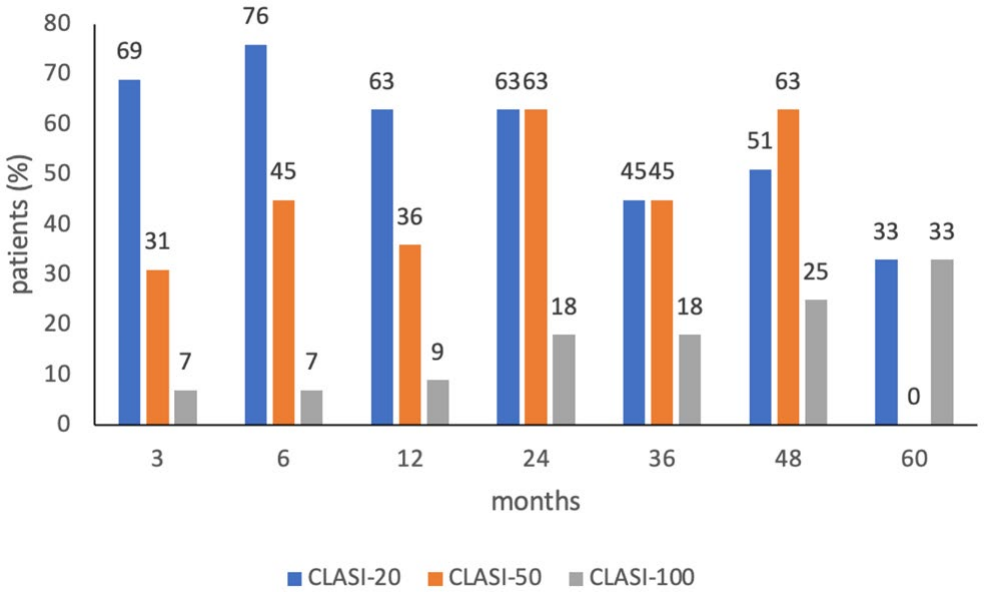
CLASI‐20, CLASI‐50, and CLASI‐100 during treatment with belimumab. Values indicate the percentage of patients achieving 20%, 50%, or 100% improvement of CLASI.

A CLASI‐20 response, indicating at least 20% reduction of CLASI, was achieved in 19 patients (64%) after 3 months, in 21 patients (72%) after 6 months, in 8 patients (72%) after 12 months, in 9 patients (81%) after 24 months, in 7 patients (63%) after 36 months, in 6 patients (76%) after 48 months, and in 4 patients (66%) after 60 months, respectively (Figure 2). A CLASI‐50 response, indicating at least 50% reduction of CLASI, was achieved in 5 patients (16%) after 3 months, in 13 patients (44%) after 6 months, in 4 patients (36%) after 12 months, in 7 patients (63%) after 24 months, in 5 patients (45%) after 36 months, in 5 patients (63%) after 48 months, and in 2 patients (33%) after 60 months, respectively (Figure 2). A CLASI‐100 response, i.e., complete remission of lupus activity in skin, was achieved in 4 patients (13%) after 3 months, in 4 patients (13%) after 6 months, in 1 patient (9%) after 12 months, in 2 patients (18%) after 24 months, in 2 patients (18%) after 36 months, in 2 patients (25%) after 48 months, and in 2 patients (33%) after 60 months, respectively (Figure 2).

**FIGURE 3:**
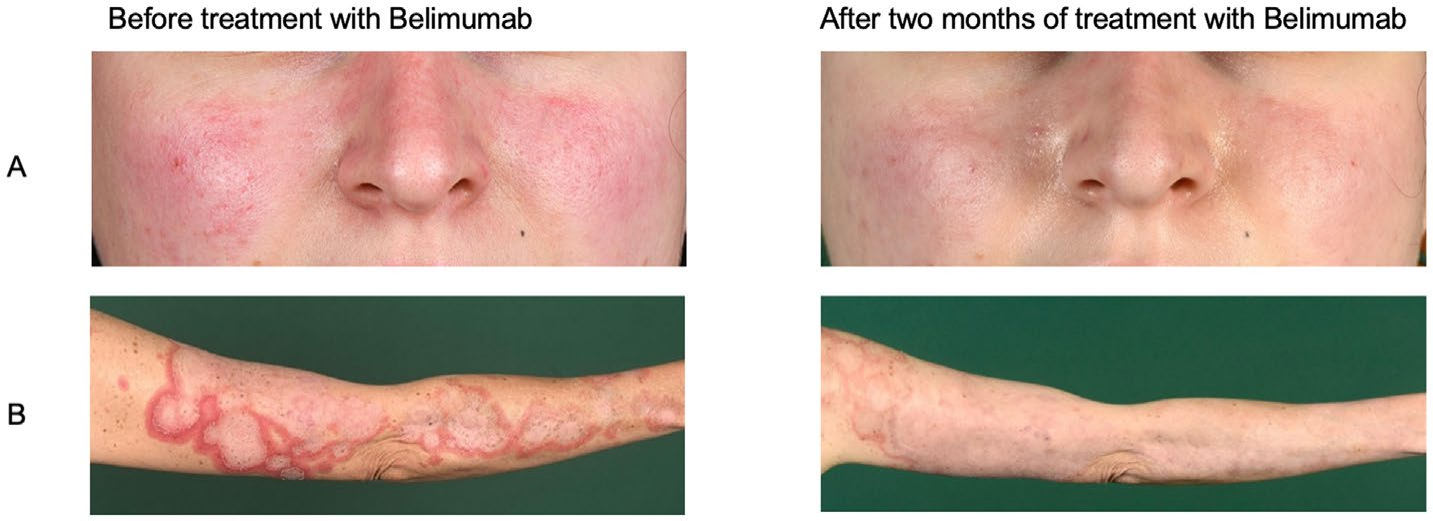
Clinical images before and after treatment with belimumab. Patient A presented with a distinct butterfly rash on her face prior to starting belimumab treatment. After 2 months of treatment with belimumab, the butterfly rash started to resolve. The right arm of Patient B showed rounded, garland‐like erythema with a central lightening and slight scaling. After 2 months of treatment with belimumab, the erythema and scaling had decreased significantly.

Examples of the clinical response in acute and subacute CLE lesions are demonstrated in (Figure 3).

**FIGURE 4:**
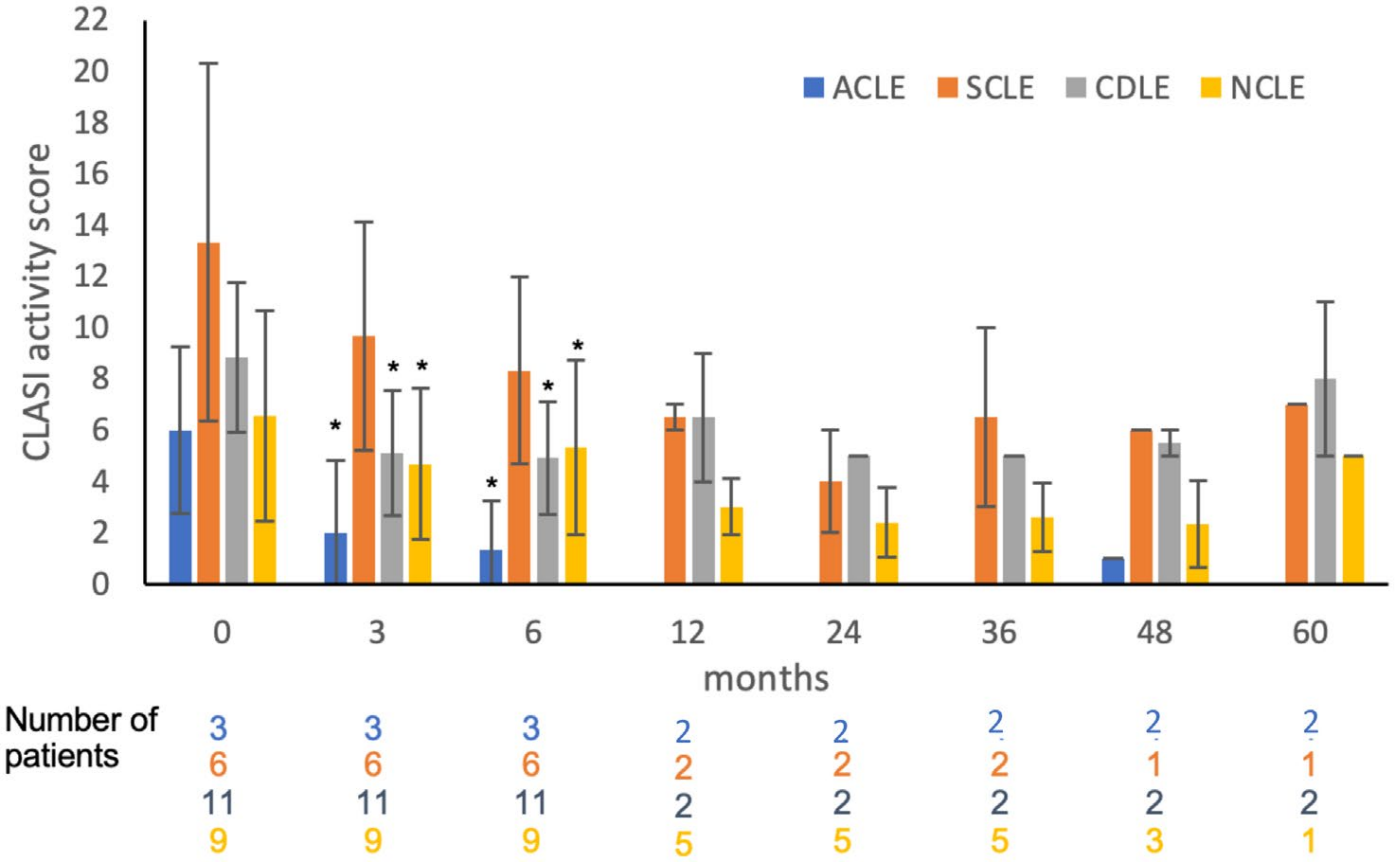
CLASI activity scores of the lupus subtypes ACLE, SCLE, CDLE, and NCLE during treatment with belimumab. ACLE—acute cutaneous lupus erythematosus, SCLE—subacute cutaneous lupus erythematosus, CDLE—chronic discoid lupus erythematosus, NCLE—non‐specific cutaneous skin lesions in lupus erythematosus. Shown are the mean and SD. Differences were tested against time point 0 before treatment and assessed by ANOVA. *p* < 0.05.

After measuring the improvement of cutaneous lupus lesions during treatment with belimumab, we asked whether subgroups of CLE responded differentially to therapy. Therefore, the CLASI and RCLASI response was assessed separately for ACLE (acute cutaneous lupus erythematosus, *n* = 3), SCLE (subacute cutaneous lupus erythematosus, *n* = 6), CDLE (chronic discoid lupus erythematosus, *n* = 11), and NCLE (non‐specific skin lesions in lupus erythematosus, *n* = 9) (Figure 4). Data show that patients with ACLE, CDLE, and NCLE had a significant reduction in CLASI activity score after 3 months (*p* = 0.018, *p* = 0.002, *p* = 0.033) and after 6 months (*p* = 0.037, *p* = 0.003, p = 0.033), respectively. In contrast, no significant reduction in CLASI activity score was observed in SCLE patients after 3 or 6 months of treatment (*p* = 0.121, *p* = 0.115). After 12 months, no significant reduction was observed in either subtype. A limitation of the analysis is the small number of patients in each subset.

### 3.2 Cutaneous BLyS Expression Is Reduced During Belimumab Therapy

**FIGURE 5:**
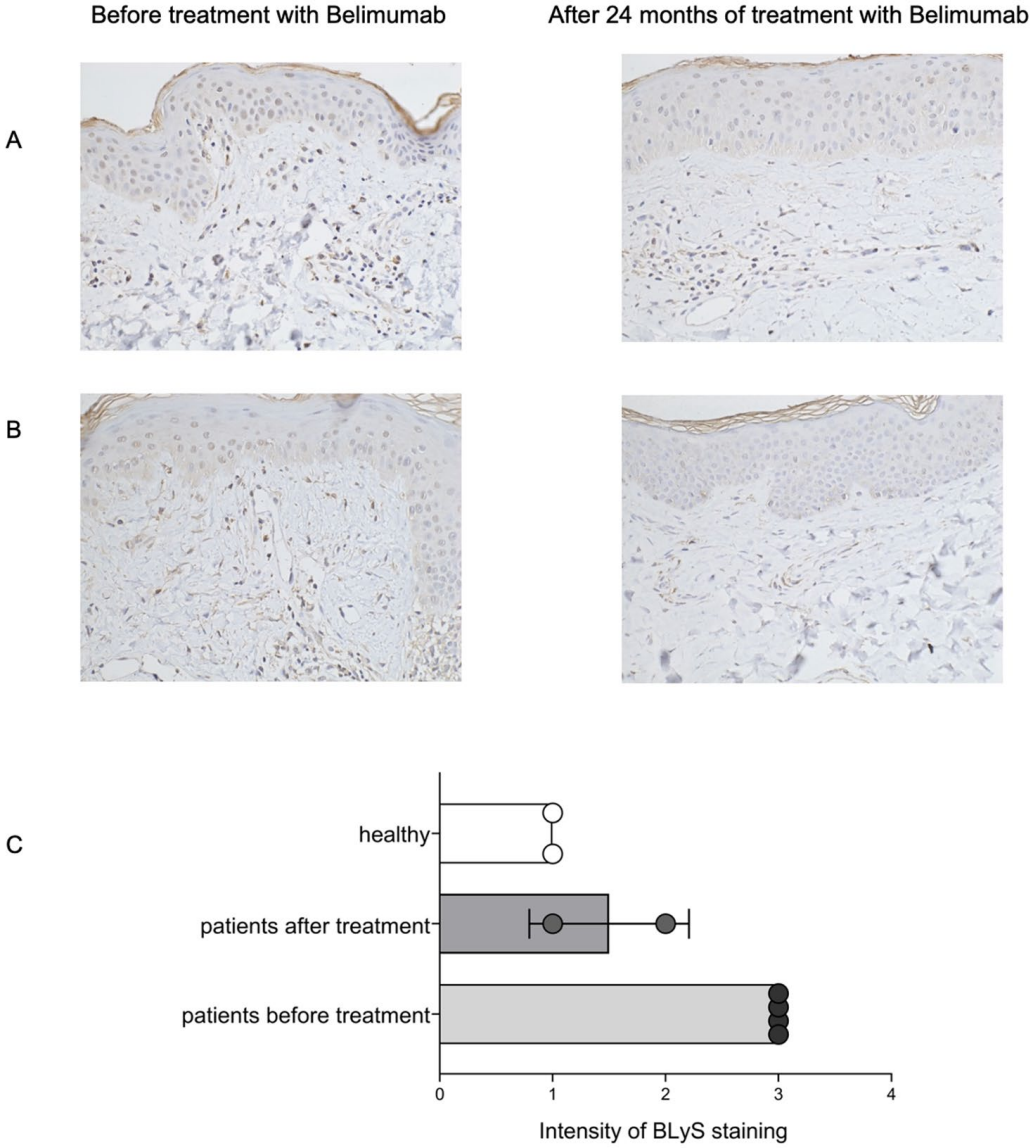
Immunohistochemical staining of BLyS. Magnification 200×. Expression of BLyS (brown) is seen in epidermis and dermis and is reduced after 24 months of treatment with belimumab. Intensity of BLyS staining is demonstrated.

Skin biopsies were available from four patients before and two patients 2 years after treatment. In each of the samples, BLyS was immunohistochemically stained. The evaluation was based on a score with 1 = weak expression of BLyS, 2 = moderate expression of BLyS, and 3 = high expression of BLyS. The skin samples of healthy subjects showed no to little expression of BLyS. In contrast, SLE skin samples exhibited obvious BLyS expression, consistent with the study by Wenzel et al. (2018) [12], which demonstrated that BLyS is not only elevated in the serum of lupus patients but also in their skin lesions. After 2 years of treatment with belimumab, the amount of BLyS detected by immunohistochemistry staining in skin was reduced (Figure 5). This suggests that BLyS expression in skin is reduced by systemic application of belimumab in lupus patients.

### 3.3 Belimumab Was Well Tolerated During Treatment

Belimumab was well tolerated by all patients included in the retrospective analysis. Eight patients had no adverse effects. Common adverse events included upper respiratory infections (*n* = 11), headache (*n* = 5), urinary tract infections (*n* = 4), and arthralgia (*n* = 4). Two patients had to pause belimumab for a short time due to respiratory infections, but were then able to continue treatment without complications. No patient had to discontinue the therapy completely due to intolerance or adverse effects. However, several patients stopped treatment due to personal reasons. One patient discontinued therapy early due to a planned pregnancy. All reported adverse events are listed in Table 2.

**TABLE 2:** Adverse events during the treatment with belimumab.

Adverse events	*n*	%
None	8/29	28
Swelling of the lip	1/29	3
Loss of appetite	2/29	7
Increase of appetite	2/29	7
Arthralgia	4/29	14
Occurrence of warts	1/29	3
Autoinflammatory syndrome	1/29	3
Vomiting	2/29	7
Exanthema	1/29	3
Queasy feeling	1/29	3
Urinary tract infection	4/29	14
Herpes zoster	2/29	7
Headache	5/29	17
Circulatory insufficiency	1/29	3
Local erythema	1/29	3
Local itching	2/29	7
Local wheals	1/29	3
Metallic taste after injection	1/29	3
Fatigue	1/29	3
Upper respiratory infection	11/29	38
Pneumonia	1/29	3
Fungal infection	2/29	7
Foetor ex ore	1/29	3
Swelling of the face, tongue	1/29	3
Vertigo	2/29	7
Nausea	1/29	3

*Note:* N = number of patients with adverse effect/total number of patients.

## 4 Discussion

Herein, we present a retrospective long‐term analysis of cutaneous lupus manifestations in patients receiving belimumab for up to 60 months in a real‐life setting at a university center. Overall, we observed a continuous and significant response of cutaneous lupus lesions under belimumab treatment. This finding aligns with previous reports demonstrating a decrease in CLASI and RCLASI activity scores after three and 6 months [13]. Iaccarino et al. also performed an analysis of the response in CLE subtypes and showed a significant reduction of the CLASI activity score in patients with ACLE and SCLE during treatment with belimumab. While our data support the finding in ACLE, the SCLE subgroup analysis did not show a significant decline during treatment. This may be related to the small number of SCLE patients analyzed in the study by Iaccarino (*n* = 5) and our analysis (*n* = 6). Interestingly, in a larger cohort, preliminary data from the same group showed a significant reduction in CLASI activity score in acute and subacute CLE after 6 months [14]. In this analysis, cutaneous involvement was limited as indicated by mean CLASI activity scores at baseline between 3.5 and 6. After 6 months, the scores declined to a mean value of 2. Further analysis did not demonstrate a further significant reduction in ACLE and SCLE, which is most likely related to the low level of cutaneous involvement. Though the sizes of the subgroups are small, they should be interpreted with caution.

The findings in this work suggest that belimumab continues to reduce skin activity even after long‐term use, which supports the study by Zen et al. (2023) [8]. Zen et al. showed that 4.5% of patients achieved CLASI remission after 6 months and 20% of patients after 24 months. Our data showed a slightly higher CLASI remission rate after 6 months of treatment, but a nearly identical percentage of CLASI remission after 36 months. We can also demonstrate that even after 60 months of treatment, 33% of patients achieved a CLASI‐100 score. The CLASI‐50 results in our work are similar to those reported by Zen et al., with our data also showing a 33% reduction in CLASI‐50 scores for patients after 60 months.

This strengthens the long‐term results of our observation. However, a limitation of our analysis is the small sample size as well as the retrospective design. More studies with a larger patient population are needed to confirm the findings in this work.

We confirmed that in the subjects from whom skin samples were taken, BLyS was elevated in lesional skin samples before treatment. Cutaneous BLyS expression had been shown previously by Wenzel et al. (2018) [12], who demonstrated that BLyS is not only elevated in the serum of lupus patients. During treatment with belimumab, the BLyS expression in skin was reduced, indicating that lesional BLyS expression might be suitable as a biomarker. Due to the small patient cohort, this finding is preliminary and should be confirmed in larger trials.

In comparison to anifrolumab, which induced fast CLASI reduction following a four‐week treatment period [15, 16], the effects of belimumab appear to be achieved at a more gradual rate. However, it is worth noting that there is currently a lack of direct comparative studies between the two treatments. Here, it was demonstrated that belimumab led to a further reduction in CLASI activity score with increasing duration of treatment. It is essential to note that long‐term data for anifrolumab are not yet available.

Deucravacitinib, a tyrosine kinase 2 inhibitor, has been shown to achieve a CLASI‐50 response in a phase II trial [17]. However, long‐term efficacy and safety have yet to be analyzed, making belimumab, as of now, a preferred option in the treatment of systemic lupus with cutaneous involvement.

Overall, belimumab was well tolerated. Common adverse events included upper respiratory infections, headache, and arthralgia, among others. In two patients, treatment was briefly paused due to adverse events but could be continued without complications after the symptoms had subsided. No patient required total discontinuation of belimumab due to side effects. The adverse event profile in this analysis was consistent with recent studies on the safety of belimumab [18, 19].

In conclusion, these real‐world data demonstrated an effective and safe long‐term improvement of the cutaneous manifestations of patients with lupus erythematosus treated with belimumab. Nevertheless, further investigation is warranted to substantiate these observations with larger sample sizes.

## Funding

This study was partially funded by a research grant from GSK awarded to Claudia Günther.

## Conflicts of Interest

C.G. received honoraria for presentations and advisory boards from GSK, AstraZeneca, Boehringer Ingelheim, Janssen, and UCB.

## Supporting information


**Figure S1:** Patient flowchart. ACLE‐ Acute Cutaneous Lupus Erythematosus. SCLE‐ Subacute Cutaneous Lupus Erythematosus. CDLE‐ Chronic Discoid Lupus Erythematosus. NCLE‐ Non‐specific skin lesions in Lupus Erythematosus.
**Table S1:** Number of evaluable patients and reasons for missing data.

## Data Availability

The data that support the findings of this study are available from the corresponding author upon reasonable request.
