# Supplementary material for: Effect of Belimumab on the Cutaneous Manifestations of Patients With Lupus Erythematosus
Source: Int J Dermatol. 2026 Jan 22;65(4):783–9. doi: 10.1111/ijd.70251 (PMC12979240; doi:10.1111/ijd.70251)
Supplement: Supplementary file 1 — Figure S1: Patient flowchart. ACLE‐ Acute Cutaneous Lupus Erythematosus. SCLE‐ Subacute Cutaneous Lupus Erythematosus. CDLE‐ Chronic Discoid Lupus Erythematosus. NCLE‐ Non‐specific skin lesions in Lupus Erythematosus. Table S1: Number of evaluable patients and reasons for missing data. [file IJD-65-783-s001.docx]

Supplementary material


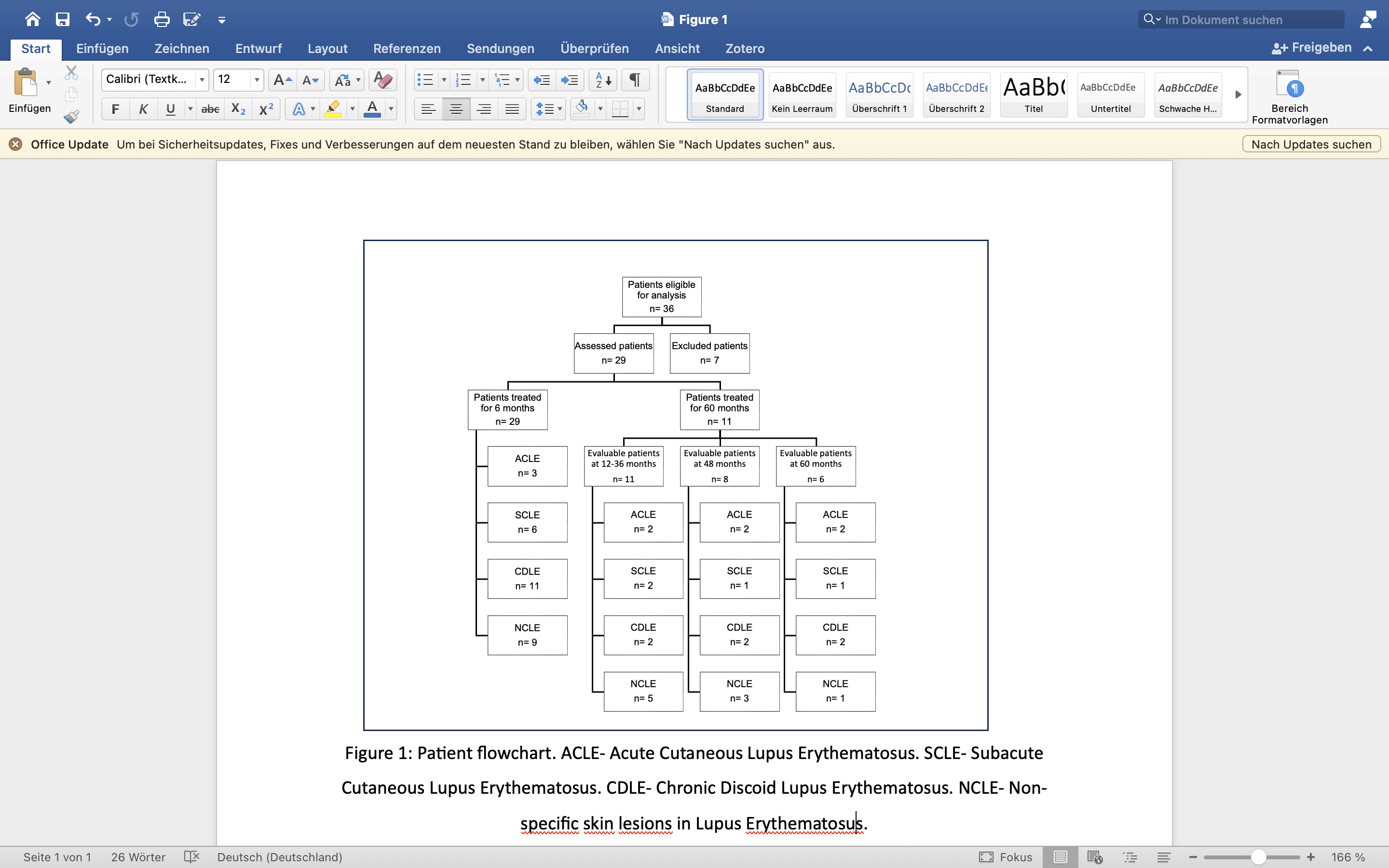


Figure S1: Patient flowchart. ACLE- Acute Cutaneous Lupus Erythematosus. SCLE- Subacute Cutaneous Lupus Erythematosus. CDLE- Chronic Discoid Lupus Erythematosus. NCLE- Non-specific skin lesions in Lupus Erythematosus.

| Month | Number of evaluable patients/treated patients N | Reasons for missing data |
| --- | --- | --- |
| 0 | 29/29 | - |
| 3 | 29/29 | - |
| 6 | 29/29 | - |
| 12 | 11/11 | - |
| 24 | 11/11 | - |
| 36 | 11/11 | - |
| 48 | 8/11 | Loss of follow-up |
| 60 | 6/11 | Loss of follow-up, intercurrent illness |

Table S1: Number of evaluable patients and reasons for missing data.
